# The association between weight loss and long term development in quality-of-life among children living with obesity: a pragmatic descriptive intervention study

**DOI:** 10.1186/s13052-022-01326-2

**Published:** 2022-07-30

**Authors:** Rasmus Møller Jørgensen, Esben Thyssen Vestergaard, Britta Kremke, Rikke Frederiksen Bahnsen, Bent Windelborg Nielsen, Jens Meldgaard Bruun

**Affiliations:** 1 Department of Pediatrics, Randers Regional Hospital, Østervangsvej 54, entrance C, 8930 Randers, NØ Denmark; 2 Steno Diabetes Center Aarhus, Aarhus University Hospital, Hedeager 3, 2nd floor, 8200 Aarhus N, Denmark; 3 Department of Clinical Medicine, Aarhus University, Aarhus, Palle Juul-Jensen Boulevard 82, 8200 Aarhus N, Denmark; 4 Department of Internal Medicine, Randers Regional Hospital, Østervangsvej 54, 8930 Randers, NØ Denmark

**Keywords:** Quality-of-life, Children, Obesity, Lifestyle intervention, Weight loss

## Abstract

**Background:**

Childhood obesity is associated with impaired Quality-of-Life (QoL), increased stigmatization and higher risk of development of depression compared to their peers. This report describes the long-term development in QoL for cohort of children with obesity after a sustainable weight reduction.

**Methods:**

This pragmatic descriptive intervention study enrolled 120 children with obesity, age 5–17 years, in a multifactorial lifestyle intervention. The intervention was an across sectors collaboration between a department of pediatrics and community health care workers. QoL was assessed yearly throughout the intervention and evaluated by a 6-item Visual Analogue Scale (VAS). For analyzing changes in VAS, as function BMI-SDS, regression models were used, while ANOVA and Wilcoxon test were applied for normal and not-normal distributed data. 95% confidence interval not containing 0 and p-value < 0.05 was considered statistically significant.

**Results:**

After 26.4 months (13.9 SD) an overall decrease in bullying (0.6 *vs.* 0.0 median) and motivation (10.0 *vs.* 9.6) was observed. QoL increased in children with a BMI-SDS reduction (0.65 (2.49 SD)) opposite children with no-change or increasing BMI-SDS who reported reduced QoL (-0.36 (1.55 SD) and -0.96 (2.27 SD)). A significant inverse relationship was observed for Joy of Life, QoL and body perception as a function of BMI-SDS per year.

**Conclusion:**

Weight reduction causes improvement in QoL for children with obesity and an inverse relationship for QoL and changing BMI-SDS / year was establish.

## Background

Children with obesity have lower Quality of Life (QoL) compared to their peers [1, 2] and an inverse relation between QoL and Body Mass Index (BMI) amongst adolescents has been reported [3]. Children with obesity also experience lower self-esteem, more bulling, and a higher degree of loneliness and risk of depression [1, 4, 5], and their QoL is comparable to that in children with chronic diseases (e.g. type 1 diabetes or cancer) [1].

The Visual Analogue Scale (VAS) can be used to evaluate QoL and is a simple instrument with good validity, excellent reliability and good responsiveness. The VAS instrument is applicable to children who have insufficient reading skills and is therefore useful in younger children [6, 7].

The literature on how to improve QoL in children with obesity is scarce, and QoL after weight loss has only been reported in a limited number of studies and rarely with follow-up for more than 12 months [8]. Recently, a 14 months multifactorial lifestyle intervention in children with obesity reported increased QoL, mood, and body satisfaction, whereas appetite, bullying, and motivation were decreased. Interestingly, similar positive changes were observed in five of six VAS items in the 24.4% of children who increased their BMI Standard Deviation Score (SDS) during the intervention, indicating that the intervention and not the weight change were responsible [8]. The aim of our project was to evaluate long-term (with a mean follow-up of > 2 years) effects of a comparable multifactorial lifestyle intervention on measures of QoL in children with obesity.

## Material and methods

### The study design

This study was designed and conducted as a municipality-based treatment for children with obesity, and therefore best described as a pragmatic descriptive intervention study, hence not a randomized study and therefore without a control group.

### Definition of obesity

In a validated Danish reference group childhood obesity is defined by an abnormal BMI-SDS or z-score (≥ 2 SD) or a BMI above the 99 percentiles [9]. BMI-SDS was calculated using GrowthXP (PC Pal, Wissous, France).

For this study, a reduction in BMI-SDS greater than -0.1 SD was considered a weight loss, while change between -0.1 to 0.1 SD or an increase of more than 0.1 SD was considered weight stagnation or weight gain, respectively.

### Subjects

One hundred and ninety-nine children with obesity [9] participated in The Children’s Obesity Clinic’s Treatment (TCOCT) protocol at the Department of Pediatrics, Randers Regional Hospital. The inclusion criteria were age 4–17 years and BMI-SDS ≥ 2SD with an inclusion period from January 1^st^ 2014 to December 31^st^ 2017. Only children without known mental retardation and syndromes associated with obesity were invited into the original intervention. One hundred and twenty children (64 girls) age 5–17 were able complete at least two VAS-questionnaires; one at baseline; and were included in this study. The participants were referred to intervention from school nurse, general practitioner and outpatient clinics. At the baseline visit, demographic data such as predispositions for obesity and mental illness were recorded at a face-to-face interview by healthcare professionals. A child was considered disposed for obesity if one or both parents had a BMI above 30 or had a bariatric surgery performed prior to baseline. If one or both parents declared that they suffered or had suffered from any kind of mental disorder, the child was considered disposed for mental illness. A parent or legal guardian provided a written informed consent before participation.

### The TCOCT protocol

TCOCT is a family-centered multifactorial lifestyle intervention involving behavioral change techniques and developed for treatment of children with obesity. At baseline, the child receives an individualized treatment plan intervening with the child and family’s daily routines and lifestyle [8]. Thereafter, children were seen once a year at our outpatient clinic and at 6–8 visits a year at community healthcare workers offices and followed for a maximum of three years in total.

At each visit at the outpatient clinic QoL would be evaluated by a six items VAS. All children were invited for a last visit and a renewed VAS in December 2018, one year after the project ended [10].

Changes in anthropometry, body composition, and biomarkers were also assessed each year and have previously been reported [11]. Psychological support was not, per se, a part of the intervention and was not offered as a routine. If necessary (e.g. clinical signs of depression) the intervention was immediately paused and the child was referred to relevant professionals.

### Assessment of QoL

VAS was used to measure participant’s well-being in six different areas: 1. Joy of living (JoL), 2. QoL, 3. Appetite, 4. Bullying, 5. Motivation for losing weight, and 6. Body perception (Bpc). All children were instructed rigorously by a healthcare professional in a uniform way to rate his/her current state of mind expressed by the six different VAS items with a single cross on a 10 cm vertical blank line, unnumbered except 0 and 10 [6, 7]. VAS and PedsQL scores have earlier been reported to correlate well down to the age of 5 years [12], and that VAS potentially could be used in even younger children, but more research is needed [13].

### Statistics

Data was collected and managed using REDCap electronic data capture tools [14]. Statistical analyses were performed in Stata 15 (StataCorp, College Station, Texas). To analyse for changes in VAS, as a function of BMI-SDS, ANOVA for normal distributed data and Wilcoxon rank-sum (2 groups) or Kruskal–Wallis (more than 2 groups) tests for non-normal distributed data were used. For categorical variables, Pearson's chi-squared test was used. 95% confidence interval not containing 0 and P-value < 0.05 was considered statistically significant.

## Results

### Baseline

The participants had a mean BMI-SDS of 3.1 (0.7 SD) and mean age of 10.4 years (2.8 SD). Approximately 90% of the participants were predisposed to obesity, half of these from both parents. Only one child was due to adoption unaware of any predispositions (Table 1). At baseline boys were more obese (mean BMI-SDS 3.3 (0.7 SD) *vs*. 2.9 (0.6 SD), *p* < 0.001) with broader mean waist circumference (93.9 cm (15.4 SD) *vs.* 87.4 (14.0 SD), *p* = 0.02), lower mean fat percentage (32.8% (6.1SD) *vs.* 37.0 (5.3 SD), *p* < 0.001) and more hungry ((mean VAS 6.7 (2.3 SD) *vs*. 5.6 (2.4 SD), *p* = 0.03) than girls. No other differences in baseline characteristics or predispositions were found between the two groups.

**Table 1:** Baseline characteristics and social conditions

	All	Boys	Girls	*p*-value
N	120	56	64	
Age, years, mean (SD)	10.4 (2.8)	10.8 (2.9)	10.0 (2.7)	0.14
BMI-SDS, mean (SD)	3.1 (0.7)	3.3 (0.7)	2.9 (0.6)	< 0.001
Waist circumference, cm, mean (SD)	90.4 (15.0)	93.9 (15.4)	87.4 (14.0)	0.02
Blood pressure systolic, mmHg, mean (SD)	118 (11.4)	119 (12.4)	117 (10.6)	0.43
TANITA, Adipose tissue percentage, mean (SD)	35.0 (6.0)	32.8 (6.1)	37.0 (5.3)	< 0.001
Disposition—overweight				0.31
One parent	49 (40.8%)	19 (34%)	30 (47%)	
Both parents	60 (50.0%)	32 (57%)	28 (44%)	
No dispositions	10 (8.3%)	4 (7%)	6 (9%)	
Unknown	1 (0.8%)	1 (2%)	0 (0%)	
Disposition—mental illness				0.14
One parent	31 (25.8%)	19 (34%)	12 (19%)	
Both parents	7 (5.8%)	2 (4%)	5 (8%)	
No dispositions	81 (67.5%)	34 (61%)	47 (73%)	
Unknown	1 (0.8%)	1 (2%)	0 (0%)	
VAS1, Joy of living, median (IQR)	9.1 (7.3, 10.0)	9.5 (7.6, 10.0)	8.8 (7.1, 10.0)	0.09
VAS2, Quality of life, median (IQR)	9.6 (7.6, 10.0)	9.6 (7.4, 10.0)	9.6 (7.8, 10.0)	0.62
VAS3, Appetite, median (IQR)	5.4 (4.6, 8.1)	6.6 (4.9, 8.9)	5.2 (4.5, 6.9)	0.03
VAS4, Bullying, median (IQR)	0.6 (0.0, 4.7)	0.4 (0.0, 4.8)	0.6 (0.0, 4.7)	0.59
VAS5, Motivation, median (IQR)	10.0 (9.0, 10.0)	10.0 (8.9, 10.0)	10.0 (9.1, 10.0)	0.57
VAS6, Body perception, median (IQR)	6.1 (3.1, 9.6)	7.8 (3.8, 9.7)	5.4 (3.0, 9.4)	0.18

*P*-values represent differences between boys and girls. Normal / not normal distributed data is reported as mean with standard deviations (SD) or median with interquartile range (IQR), while categorical variables are reported as n with percentage (%)

Throughout the trial 81 children (68.5%) achieved a BMI-SDS reduction (< -0.1 ∆BMI-SDS), 19 children (15.8%) experienced no change (-0.1–0.1 ∆BMI-SDS) and 20 children (16.7%) increased BMI-SDS (> 0.1 ∆BMI-SDS). There were no demographic or anthropometric differences at baseline between the three BMI-SDS groups (Table 2).

**Table 2:** Baseline characteristics for weight development

Weight developement	Loss	Stag	Gain	*p*-value
N	81	19	20	
Age, years, mean (SD)	10.1 (2.7)	11.2 (2.7)	10.9 (3.2)	0.19
BMI-SDS, mean (SD)	3.1 (0.7)	3.1 (0.6)	2.9 (0.7)	0.64
Sex				0.30
Boys	34 (61%)	10 (19%)	12 (21%)	
Girls	47 (73%)	9 (14%)	8 (13%)	
Waist circumference, cm, mean (SD)	89.3 (14.9)	96.7 (14.6)	88.9 (14.7)	0.14
Blood pressure systolic, mmHg, mean (SD)	118 (11.0)	121 (14.0)	115 (10.2)	0.28
TANITA, Adipose tissue percentage, mean (SD)	34.8 (6.2)	36.8 (6.2)	34.3 (5.3)	0.36
VAS1, Joy of living, median (IQR)	9.1 (7.3, 10.0)	9.8 (6.9, 10.0)	9.0 (7.6, 10.0)	0.84
VAS2, Quality of life, median (IQR)	9.4 (7.0, 10.0)	9.6 (8.2, 10.0)	10.0 (8.0, 10.0)	0.41
VAS3, Appetite, median (IQR)	5.7 (4.7, 8.1)	5.3 (4.2, 7.2)	5.5 (4.9, 8.9)	0.58
VAS4, Bullying, median (IQR)	1.0 (0.0, 4.7)	0.4 (0.0, 5.1)	0.2 (0.0, 4.7)	0.55
VAS5, Motivation, median (IQR)	10.0 (9.0, 10.0)	10.0 (9.7, 10.0)	10.0 (8.1, 10.0)	0.77
VAS6, Body perception, median (IQR)	5.4 (3.0, 9.6)	6.9 (3.2, 9.4)	8.1 (3.7, 10.0)	0.39

Weight development are divided into weight loss (< -0.1 ∆BMI-SDS), weight stagnation (-0.1 to 0.1 ∆BMI-SDS) and weight gain (> 0.1 ∆BMI-SDS). Normal / not normal distributed data is reported as mean with standard deviations (SD) or median with interquartile range (IQR), while categorical variables are reported as n with percentage (%). P-values refer to comparisons between the variables in each group

### Changes in VAS:

Mean time between the first and last VAS measurement was 26.4 months (13.9 SD). On average 2.7 VAS measurements were completed for each participant.

As outlined in Table 3, a significant decrease in bullying (0.6 median (0.0–4.7) IQR *vs.* 0.0 median (0.0–0.6) IQR, *p* < 0.001), and in motivation (10.0 median (9.0–10.0) IQR *vs.* 9.6 median (7.0–10.0) IQR, *p* = 0.002), was observed. No significant changes were found for JoL, QoL, appetite, or Bpc.

**Table 3:** The relative development for the 6 VAS scores^a^ for all participants, gender and weight development

	Overall			Gender			Weight development			
	All	*p*-values*	Boys		Girls	*p*-values	Loss	Stag	Gain	*p*-values
N	120		56		64		81	19	20	
∆VAS1, Joy of living	0.37 (2.12)	0.51 *	-0.04 (2.10)		0.73 (2.08)	0.048	0.63 (1.98)	-0.01 (2.39)	-0.31 (2.26)	0.14
∆VAS2, Quality of life	0.22 (2.40)	0.60 *	0.32 (2.61)		0.13 (2.22)	0.67	0.65 (2.49)	-0.36 (1.55)	-0.96 (2.27)	0.01
∆VAS3, Appetite	-0.46 (3.06)	0.14 *	-0.98 (3.22)		-0.01 (2.86)	0.08	-0.45 (3.10)	-0.17 (2.89)	-0.78 (3.14)	0.82
∆VAS4, Bullying	-1.40 (3.18)	< 0.001 *	-1.23 (3.30)		-1.56 (3.10)	0.57	-1.69 (3.16)	-0.93 (3.75)	-0.67 (2.66)	0.34
∆VAS5, Motivation	-0.78 (2.90)	0.002 *	-1.02 (2.93)		-0.57 (2.87)	0.40	-0.63 (2.90)	-1.41 (2.36)	-0.81 (3.37)	0.58
∆VAS6, Body perception	0.69 (3.69)	0.27 *	0.29 (3.66)		1.03 (3.71)	0.28	1.14 (3.45)	0.49 (3.82)	-0.96 (4.17)	0.07

The last category is sub grouped in weight loss (< -0.1 ∆BMI-SDS), weight stagnation (-0.1 to 0.1 ∆BMI-SDS) and weight gain (> 0.1 ∆BMI-SDS)Ұ. P-values refer to comparisons between the variables in each group (respectively gender and weight development). All data is reported as mean value with standard deviations (SD)

Ұ∆BMI-SDS was calculated as the difference between BMI-SDS at baseline and BMI-SDS at the latest obtained VAS

^a^The relative development for VAS (∆VAS) was calculated as the difference between baseline and at the latest obtained VAS

^*^These *p*-values refer to comparison between the baseline VAS (Table1) and the last VAS obtained (not shown)

Stratified by sex, boys experienced decreased mean appetite (6.7 (2.3 SD) vs. 5.7 (2.6 SD), *p* = 0.04), bullying (0.4 median (0.0–4.8) IQR *vs.* 0.0 median (0.0–0.8) IQR, *p* = 0.03), and motivation (10.0 median (8.9–10.0) IQR *vs.* 8.9 median (6.6–10.0) IQR, *p* = 0.003). Girls experienced decreased bullying (0.6 median (0.0–4.7) IQR *vs.* 0.0 median (0.0–0.6) IQR, *p* < 0.001).

Children who achieved a weight loss (< -0.1 ∆BMI-SDS) experienced reduced bullying (1.0 median (0.0–4.7) IQR *vs.* 0.0 median (0.0–0.6) IQR, *p* < 0.001) and motivation (10.0 median (9.0–10.0) IQR *vs.* 9.6 median (7.3–10.0) IQR, *p* = 0.027). Children without change in BMI-SDS (-0.1 to 0.1 ∆BMI-SDS) experienced loss of motivation (10.0 median (9.7–10.0) IQR *vs.* 9.7 median (5.7–10.0) IQR, p = 0.046). No change was observed for those with increased BMI-SDS (data not shown).

### Relative changes in VAS

Comparing the relative changes in VAS for boys and girls, mean JoL in girls increased significantly ((0.73 (2.08 SD) girls) *vs.* (-0.04 (2.10 SD) boys), p = 0.048).

Children with reduced BMI-SDS increased their mean QoL (0.65 (2.49 SD)), compared to no change BMI-SDS (-0.36 (1.55 SD)) or increased BMI-SDS (-0.96 (2.27 SD)), *p* = 0.01 (Table 3). Reducing BMI-SDS also increased mean JoL and mean Bpc, although non-significant (*p* = 0.14 and *p* = 0.07, respectively, Table 3), compared with children with increasing BMI-SDS.

For the last visit, regression analyses showed similar results, when investigating the association between the relative change in VAS as a function of the relative change in BMI-SDS over time (years). We here observed a significant inverse association for VAS1 JoL (-1.57 (0.74 SD) CI (-3.02 to -0.10)) VAS2 QoL (-1.71 (0.84 SD) CI (-3.38 to -0.05)) and VAS6 body perception (-2.78 SD 1.29 CI (-5,31 to -0.22)). Non-significant tendencies were observed for bullying (2.02 (1.15 SD) CI (-0.19 to 4.23)) and motivation (-1.94 (1.01 SD) (-3.95 to 0.68)).

## Discussion

The aim of the study was to evaluate different measures of QoL after a lifestyle intervention in children with obesity. With a mean follow-up of 26.4 months, this is to our knowledge the longest follow-up on QoL after a weight loss intervention.

An overall reduction in bullying and motivation was observed. Boys experienced a reduction in appetite, bullying and motivation, while girls experienced reduced bullying and improved JoL.

Interestingly, QoL was significant improved for children who reduced BMI-SDS compared to children with no-change or increasing BMI-SDS after the intervention. Similar but non-significant trends were observed for JoL, bullying, and Bcp. The weight development in BMI-SDS (reduction, no change, or increase, respectively) and the concomitant dose-dependent changes in QoL is to our knowledge not previously reported. A significant inverse relationship was observed for VAS1 JoL, VAS2 QoL and VAS6 body perception as a function of BMI-SDS per year. This underlines that a greater reduction BMI-SDS / year is associated with improvement in several aspects of quality of life (JoL, QoL and body perception) for children living with obesity.

It is thoroughly described that obesity in childhood is associated with imminent risk of decreased self-esteem, self-perception and QoL [1, 8, 15]. Strauss et al. suggest that lower self-esteem in children with obesity is associated with increasing levels of sadness, loneliness, and anxiety [15], while Paxton et al. predict that decreased body satisfaction in time will lead to depressive moods and low self-esteem [16]. In addition, children and adolescents with obesity are more likely to be the victim of bullying [4], which might aggravate the negative effects of living with obese. The current study indicates that some of these negative effects on mental health is not necessarily permanent, but reversible condition associated with weight reduction, where a greater reduction in BMI-SDS / year is associated with greater improvement in the mental health condition (JoL, QoL and body perception) in children living with obesity.

Using VAS for younger children (< 6 years of age) might challenge the child's understanding of the questions and the child’s perception of the questions might change with change in age. In a cross-sectional study assessing QoL for children with oral cleft, VAS and PedsQL scores correlated well (r = 0.67) for children 5 to 10 years of age [12]. In the current study we included four children 5 years of age, but excluded children not able to complete the VAS—regardless of age. We didn't find literature on age-depending changings in perception of VAS for children living with obesity.

One of the strengths is that all VAS scores were obtained in uniformed method with simple instructions from a small team of specifically trained healthcare professionals.

An obvious limitation is the lack of a control group. In addition, the limited number of participants increased the risk of type-2-error, exemplified by the borderline-significant results.

The main purpose of the intervention is to reduce BMI-SDS, however, it was considered an equal success to improve QoL and thereby reduce bullying and the development of psychosocial complications such as the risk of depression later in life [1, 4, 5].

The present intervention is broadly used in the treatment of children with obesity in Denmark and our results ad to previous findings [8], by including long-term follow-up in children who were more obese and younger. Fonvig et *al.* reported beneficial changes in 5 out of 6 VAS-items for children with increasing BMI-SDS [8]. Similar changes in QoL after gaining weight during an intervention have been reported elsewhere, but with shorter follow-up of 4 months and 4 weeks, respectably [17, 18]. In contrast, our intervention displayed children with increasing BMI-SDS to have impaired JoL, QoL, and Bcp. The discrepancies may be explained by the longer follow-up in perhaps a more real-life situation and that the beneficial effects of lifestyle intervention on QoL lasts shortly if not accompanied by a weight reduction.

## Conclusion

Weight reduction cause improvement in QoL for children with obesity and a significant inverse relationship was observed for JoL, QoL and body perception as a function of BMI-SDS changes per year.

## Data Availability

The data used during the current project are available from the corresponding author on reasonable request.

## Abbreviations

QoL: Quality-of-Life
BMI: Body mass index
VAS: Visual Analogue Scale
SDS: Standard deviation score
TCOCT: The Children’s Obesity Clinic’s Treatment
JoL: Joy of living
Bpc: Body perception
